# Sepsis causes right ventricular myocardial inflammation independent of pulmonary hypertension in a porcine sepsis model

**DOI:** 10.1371/journal.pone.0218624

**Published:** 2019-06-27

**Authors:** Soeren Erik Pischke, Siv Hestenes, Harald Thidemann Johansen, Hilde Fure, Jan Frederik Bugge, Andreas Espinoza, Helge Skulstad, Thor Edvardsen, Erik Fosse, Tom Eirik Mollnes, Per Steinar Halvorsen, Erik Waage Nielsen

**Affiliations:** 1 Department of Immunology, University of Oslo and Oslo University Hospital, Oslo, Norway; 2 Department of Anaesthesiology, Division of Emergencies and Critical Care, Oslo University Hospital, Oslo, Norway; 3 Faculty of Medicine, University of Oslo, Oslo, Norway; 4 Intervention Centre, Oslo University Hospital, Oslo, Norway; 5 Department of Anaesthesia, Intensive Care and Emergency Medicine, Vestre Viken Baerum Hospital, Baerum, Norway; 6 Department of Pharmaceutical Biosciences, School of Pharmacy, University of Oslo, Oslo, Norway; 7 Research Laboratory, Nordland Hospital, Bodø, and Faculty of Health Sciences, K.G. Jebsen TREC, University of Tromsø, Tromsø, Norway; 8 Department of Cardiology, Oslo University Hospital and University of Oslo, Oslo, Norway; 9 Centre of Molecular Inflammation Research, Norwegian University of Science and Technology, Trondheim, Norway; Max Delbruck Centrum fur Molekulare Medizin Berlin Buch, GERMANY

## Abstract

**Introduction:**

Right ventricular (RV) myocardial dysfunction is a common feature in septic shock. It can worsen outcome, but the etiology is poorly understood. Pulmonary artery hypertension (PAH) plays a part in the pathogenesis of the right heart dysfunction in sepsis but its importance is unknown. In pigs, PAH in sepsis is substantial and the translational value of porcine sepsis models therefore questioned. We hypothesized that porcine sepsis causes a myocardial inflammatory response which leads to myocardial dysfunction independent of PAH.

**Materials and methods:**

Sepsis was induced by *Escherichia coli*-infusion in 10 pigs resulting in PAH and increased right ventricular pressure (RVP). The same degree of RVP was achieved by external pulmonary artery banding (PAB) in a consecutive series of 6 animals.

**Results:**

Sepsis, but not PAB, led to increase in endothelial damage marker PAI-1 and cytokines TNF and IL-6 (all p<0.05) in plasma. In myocardium, TNF and IL-6 were significantly elevated in sepsis, TNF in both ventricles and IL-6 mostly in RV, while IL-1β, IL-18 and C5a were significantly higher in RV compared to LV after PAB (all p<0.05). Myocardial mRNA levels of IL-1β, IL-6, IL-18, IP-10, E-selectin and PAI-1 were significantly elevated in RV and LV during sepsis compared to PAB, while Caspase-1 was decreased in septic compared to PAB animals (all p<0.05). Cathepsin L activity was increased in RV by PAB, while sepsis inhibited this response. *Escherichia coli*-induced sepsis caused myocardial inflammation independent of PAH.

**Conclusion:**

Prominent PAH should therefore not exclude porcine sepsis models to further our understanding of human sepsis.

## Introduction

Sepsis is a leading cause of mortality and admission to intensive care world-wide. Investigations of human sepsis in a clinical setting are obscured by numerous covariate factors, especially co-morbidities [1]. To reduce these factors and still simulate human sepsis, porcine models are often preferred [2].

Compared to rodent models, porcine models have comparable endotoxin sensitivity and tissue antigenicity to humans, similar cardiovascular and renal physiology and a size allowing for instrumentation and monitoring with equipment in daily clinical use [3][4]. Additionally, the possibility of serial blood sampling combined with powerful repeated measurements statistics can result in significant findings with low animal numbers [5]. Even porcine models of neonatal sepsis have been developed for the same reason [6].

Acute myocardial depression and dysfunction is frequent in sepsis. Ventricular dysfunction has been observed in up to 60% of adult septic shock patients within the first 3 days and affects global heart function [7]. Pathology in various Ca^2+^ transporters and myofilaments has been identified in animal studies. However, the underlying cause of the dysfunction remains elusive [8], and further investigations are warranted [9]. Right ventricular (RV) dysfunction and dilation is multifactorial and might be predictive of clinical outcome [10][11]. It can be induced by afterload increase due to pulmonary hypertension (PAH), positive pressure ventilation [12], and due to direct effects of inflammatory cytokines on the myocardium [13]. In addition, lysosomal cysteine proteases like cathepsins B and L may take part in septic cardiomyopathy as they have been implicated in cardiac injury and remodeling [14]. Cathepsin B and L were recently also proposed as markers of dilated cardiomyopathy [15].

Compared to humans, sepsis in pigs triggers a much larger rise in pulmonary vascular resistance, resulting in a very dominant PAH. PAH in pigs can to a large extent be explained by large amounts of macrophages in the porcine lung parenchyma reaching 14 x 10^3^ mm^-3^ [16]. The macrophage densities are similar to that of Kupfer cells in the rat liver [16].

The effects of sepsis-induced PAH on myocardial inflammation in pigs is unknown. We hypothesized that an increase of pro-inflammatory mediators in the myocardium is due to sepsis and not due to PAH. Therefore, we compared *Escherichia coli* (*E*. *coli*) induced septic PAH to mechanical right heart stress obtained by pulmonary artery banding with an external occlusion device encircling the pulmonary trunk in pigs and evaluated differences between global and myocardial inflammatory mediators in the two groups.

## Material and methods

### Animal preparation

The study was approved by the National Animal Research Authority (Authorization Number 92/08-1532) and animals were treated and are reported in accordance to ARRIVE guidelines (see S1 Table, which describes all items of the ARRIVE guideline). In total, 16 Norwegian Landrace pigs (mean weight; 52 ± 3 kg, female:male; 12:4) were included in the study. 10 animals received *E*. *coli*-induced sepsis and 6 animals received pulmonary artery banding. The group with *E*. *coli-*induced sepsis are reported in a different study investigating strain echocardiography in detecting myocardial dysfunction in severe sepsis, the animals in this study were handled as described in the study protocol [17]. Briefly, anesthesia was induced by intramuscular ketamine (1500 mg), atropine (1 mg), and azaperone (160 mg), deepened with intravenous (iv) pentobarbital (3–5 mg/kg) during airway management and then maintained throughout protocol with iv morphine infusion (0.1–0.3 mg kg^-1^ h^-1^) and inspired isoflurane (1.5% during surgery, 0.6% thereafter) in oxygen/air mixture. After sternotomy, the middle of the RV was punctured and a micromanometer (Millar Instruments, Houston TX) inserted. Quality of the pressure curve was evaluated before each measurement and the micromanometer repositioned if necessary. An inflatable vascular occluder (In Vivo Metric, Healdsburg, CA, USA) was placed around the pulmonary artery (PA) and it was confirmed that the deflated occluder did not alter RV pressure. Regulation of the grade of pulmonary artery banding was achieved using a manually adjustable syringe pump.

### Experimental protocol

After a 30 min stabilization period following surgery, experimental sepsis was induced by increasing (every 30 minutes) doses of heat-inactivated (60 minutes at 60°C) *E*. *coli* bacteria (strain LE392, American Type Culture Collection, VA, USA, grown as described previously [18]). Bacteria were diluted in isotonic saline to a total amount of 1.5 × 10^9^ bacteria and given during the 240 minutes of the study period, this number is previously described to induce severe septic response [19].

After the first 10 sepsis animals in the study, the peak systolic right ventricular pressures were collected from the micromanometer catheter in the RV. Peak systolic RV pressures were calculated and the average used in the 6 consecutive animals to guide degree of pulmonary artery banding during the 240 minutes’ study period. Protocol excluded use of vasoactive drugs or inotropes in order not to interfere with cardiac function and dynamics.

Arterial blood samples were obtained at baseline, after stabilization prior to induction of sepsis or pulmonary artery banding, and at 60, 120, and 240 minutes after induction. Heparinized blood was used for blood gas analysis, using a ABL800FLEX blood gas analyzer (Radiometer, Brønshøj, Denmark). Citrate- and EDTA-blood were immediately cooled and centrifuged at 2500 x g for 15 min prior to storage at -80°C. The volume of the blood samples collected for use was 60 ml in total for each animal.

Pigs were euthanized at the end of the experiments, as described in S1 Table. Samples from the RV, LV and lung were immediately obtained, rinsed in ice-cold saline and snap-frozen on dry-ice prior to storage at -80°C.

### Echocardiography

The echocardiography method is explained in detail in Hestenes et al [17]. Briefly, echocardiography was carried out directly on the heart using a M4S transducer on a Vivid 7 ultrasound scanner (GE Vingmed Ultrasound, Horten, Norway) and postprocessed using EchoPac Software (GE Vingmed Ultrasound, Horten, Norway). Right ventricular (RV) function was assessed as tricuspid annular plane systolic excursion (TAPSE) and peak systolic RV strain as described previously [17]. RV strain was assessed as mean of basal, mid, and apical segments on the lateral wall over three heart-beats. Data for the sepsis animals has been presented previously [17], while data for the PAB animals is presented in this study only. Data was assessed by the same observer.

### Quantitation of protein markers of inflammation and coagulation

Coagulation markers plasminogen activator inhibitor-1 (PAI-1) and thrombin-antithrombin complex (TAT) were assessed in citrate plasma. Inflammation markers tumor necrosis factor (TNF) and interleukin (IL)-6 were assessed in EDTA plasma. Cytokines TNF, IL-1β, IL-6, IL-8, IL-18 and anaphylatoxin C5a were measured in whole protein tissue extracts. Tissue extracts were prepared as previously described [19]. Commercial ELISA was used for all analysis according to manufacturer’s instructions. Quantikine porcine immunoassay kits from R&D Systems (Minneapolis, MN) were used for analyses of TNF, IL-1β, IL-6, and IL-8. IL-18 and C5a were analyzed using kits from Uscn Life Science inc, Wuhan, China. TAT complexes were measured with a human enzyme immunoassay kit (Dade Behring, Marburg, Germany), which detects porcine TAT [20]. Plasminogen activator inhibitor-1 (PAI-1) was measured by a porcine PAI-1 activity assay kit (Molecular Innovations, Novi, MI, USA).

### Quantitative real-time polymerase chain reaction analysis

Total RNA, free of contaminating DNA, was prepared using Trizol Reagent (Invitrogen, Thermo Fisher Scientific, Waltham, MA), RNeasy MinElute Cleanup kit (Qiagen, Hilden, Germany) and subsequent DNAse treatment (Thermo Fisher Scientific, Waltham, MA) as described previously [19]. RNA quantity was assessed with NanoDrop 2000. A number of samples were tested for quality in an Agilent 2100 BioAnalyzer (Agilent Technologies, Santa Clara, CA), giving a mean RNA integrity index (RIN) of 9.5. Synthesis of cDNA was done using random primers and High-Capacity cDNA Reverse Transcription Kit (Thermo Fisher Scientific, Waltham, MA). Amount of input RNA was 500 ng in a volum of 50 μl. Cycling conditions were set to 120 min of reverse transcription at 37°C and 5 s at 85°C to stop the reaction. qPCR was run in triplicates for each candidate gene in a 15 μl reaction volume (5 ng of cDNA, 0.75 μl TaqMan Gene Expression Assay Mix, 7.5 μl TaqMan Fast Universal PCR Master Mix, and 6.25 μl nuclease-free water) in MicroAmp Fast 96-Well Reaction Plates (all reagents from Thermo Fisher Scientific). PCR amplification was performed in a 7500 Fast Real-Time PCR System instrument (Thermo Fischer Scientific) with standard cycling conditions (95°C for 20 s, followed by 40 two-segment cycles at 95°C for 3 s and at 60°C for 30 s). Predeveloped TaqMan gene expression assays (all from Thermo Fisher Scientific) for the following candidate genes were used; IL-1β (Ss03393804_m1), IL-6 (Ss03384604_u1), IL-18 (Ss03391204_m1), PAI-1 (Ss03392656_u1), E-Selectin (Ss03394520_m1), caspase-1 (Ss03394227_m1), and Interferon-γ induced protein (IP)-10 (Ss03391846_m1). Ribosomal Protein S 18 (Ss03391031_g1) was used as endogenous control and was stably expressed in all samples. The relative quantification of mRNA expression was calculated using the comparative Ct (ΔΔCt) method.

### Activities of cysteine proteases in tissue homogenates

Tissue homogenates were prepared from frozen samples of myocardial and lung tissue weighing from 30 to 60 mg. Lysis buffer was added to a final concentration of 5% weight/volume (tissue weight/total volume) (100 mM sodium citrate, 1 mM disodium-EDTA, 1% n-octyl-β-D-glucopyranoside at pH 5.8; Merck, Kennilworth, NJ). The samples were homogenized in a Precellys24 according to the manufacturer (Bertin Corp., Rockville, MD) at 5000 rpm in 50 seconds, repeated 4 times with the homogenate kept on ice between runs to avoid heating. The homogenates were centrifuged at 10,000 x g for 5 min at 4°C, and the supernatant frozen at -70^○^C for further analysis.

Legumain activity was measured by the cleavage of substrate Z-Ala-Ala-Asn-AMC (Department of Biochemistry, University of Cambridge, UK) as previously described [21][22]. Tissue homogenates (20 μl) were added to black 96-well microplates (No. 3915; Costar, Corning, NY, USA). After the addition of 100 μl buffer and 50 μl substrate solution (final concentration 10 μM), a kinetic measurement based on increase in fluorescence over 10 min was performed. Temperature was kept at 30°C and all measurements were performed in triplicate. Cathepsin B activity was measured in a similar manner except for the use of the substrate Z-Arg-Arg-AMC (20 μM; Bachem AG, Bubendorf, Switzerland) and a buffer system described elsewhere [23]. Cathepsin L activity was measured using the substrate Z-Phe-Arg-AMC (40 μM; Bachem AG) in the presence of the cathepsin B-specific inhibitor CA074 (0.25 μM; Merck, Kennilworth, NJ) as previously reported [23], with and without the addition of the cathepsin L-specific inhibitor Z-Phe-Tyr(t-Bu)-diazomethylketone (2.5 μM; Calbiochem).

### Data presentation and statistical analysis

Sample size in this sequential, non-randomized study was calculated on a basis of myocardial function as reported by Hestenes et al. [17] for septic and pulmonary artery banding animals. As septic animals were expected to be significantly more heterogeneous than pulmonary artery banding animals, 10 animals in the septic group and 6 animals in the pulmonary artery banding group were included in this study.

In two cases, circulatory collapse with subsequent death occurred at 180 min and in one case at 120 min after *E*. *coli* infusion was started. Thus, continuously reported data include nine animals at 180 min and seven animals at 240 min in the sepsis group. All animals in the pulmonary artery banding group were included at all time-points.

Pulmonary artery banding affects predominately the RV. Thus, we decided out of an ethical viewpoint regarding the 3R’s of animal research (EU directive 2010/63/EU) that the LV in pulmonary artery banding animals should serve as a negative control and did not include a group of sham treated animals. Therefore, tissue specimens were taken solely from free ventricle walls and not from the septum, as the septum most probably was affected by pulmonary artery banding. Additionally, we analyzed lung tissue for all protein parameters assessed in this study and showed that only sepsis induced by *E*. *coli* infusion induced significant changes in lung tissue, which is the tissue between the right and left ventricle (see S1 Fig which illustrates the effect of *E*. *coli* infusion on lung tissue inflammation in comparison to pulmonary artery banding).

All data are reported as median (interquartile range) except for qPCR data, which is reported as mean ± 95% confidence intervals. Continuous data was evaluated by generalized linear mixed model (subject as random effect, treatment as fixed effect) with post-hoc comparison to baseline and pairwise comparison between pulmonary artery banding and sepsis at each time-point. Data obtained at specific time-points was compared using a Mann-Whitney U test. qPCR results were compared with a 1-way ANOVA test. Post-hoc multiple comparisons between RV and LV as well as between sepsis and pulmonary artery banding were Bonferroni corrected. A p < 0.05 was considered statistically significant. Statistical analyses were performed using SPSS 22 (IBM, Armonk, NY) and graphs were prepared with GraphPad Prism 6 (GraphPad Software, La Jolla, CA).

## Results

**Fig 1:**
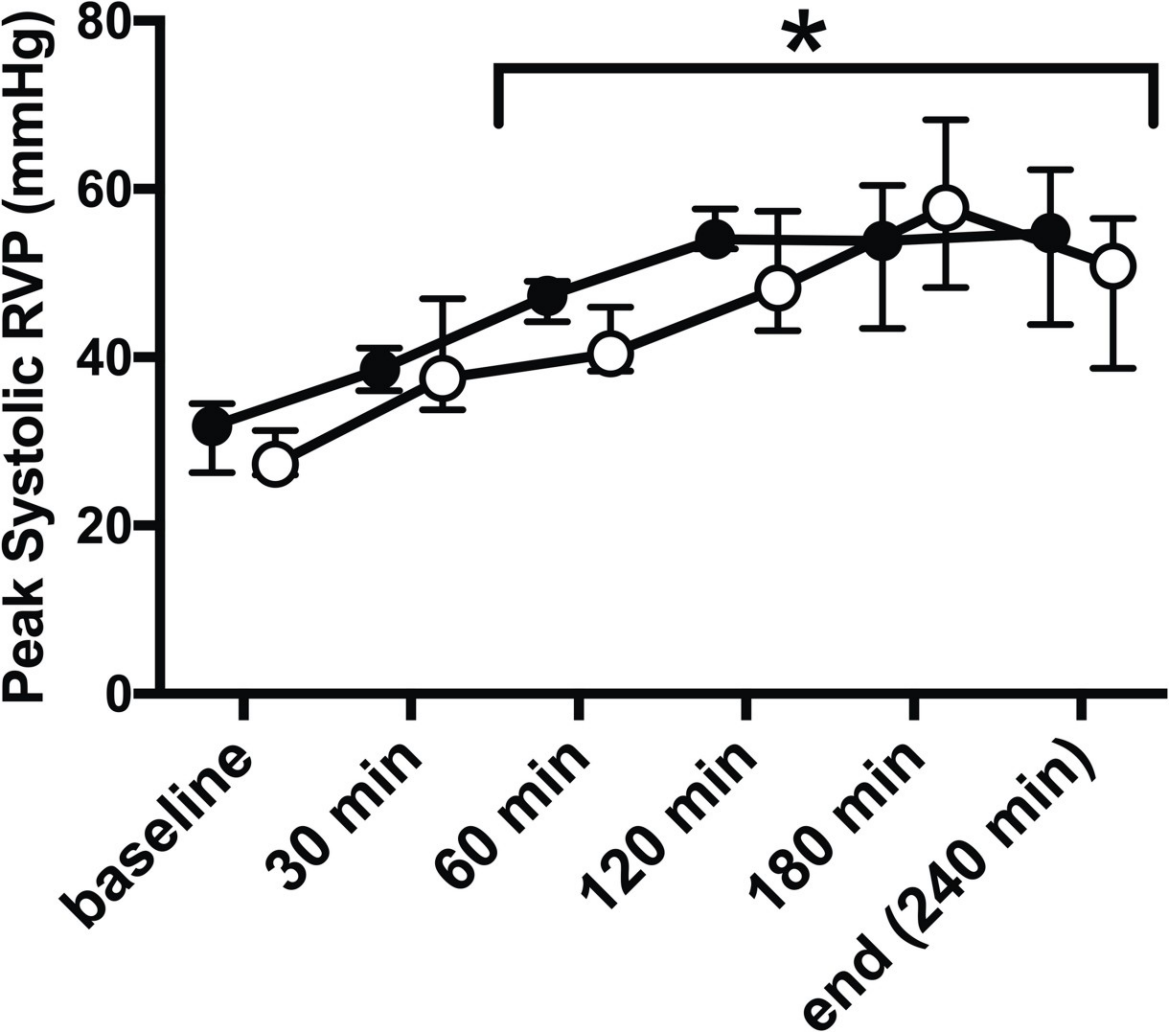
E. coli infusion increases RV pressure, which was mimicked by pulmonary artery banding. Infusion of *E*. *coli* (filled circles) caused significant increase of peak systolic right ventricular pressure (RVP) assessed by RV manometer surveillance at 60 min and throughout the remaining study period (*, p < 0.01). Manual external pulmonary artery banding (open circles) mimicked these changes with similar and significant RVP increase after 30 min and was at no time-point during the study significantly different from *E*. *coli* induced peak systolic RVP. All values Median ± Interquartile Range. Generalized linear mixed model with post-hoc comparison to baseline and pairwise comparison between pulmonary artery banding and sepsis at each time-point. Bonferroni correction for multiple testing.

Infusion of *E*. *coli* significantly increased peak systolic RVP from baseline (29 (27;32) mmHg) after 30 min of infusion (p = 0.017) with continuous significant elevation throughout the experiment (51 (42;55) mmHg, p = 0.001, Fig 1). This increase was mimicked using external pulmonary artery banding. Peak systolic RVP was at no time-point significantly different between the animals with sepsis or pulmonary artery banding (Fig 1).

Infusion of *E*. *coli* led to disturbance of systemic hemodynamics and global heart function corresponding to changes seen in sepsis. Sepsis pigs received significantly more crystalloids than control animals in order to maintain hematocrit and blood pressure (19 (17;20) L vs. 9 (8;10) L, p = 0.003). Septic animals showed a significant increase in heart rate, mean pulmonary artery pressure, lactate, and a decreased left ventricular stroke volume in comparison to baseline, while right heart function assessed as right ventricular strain was unchanged and assessed as TAPSE decreased after 240 minutes only (Table 1). Contrary, pulmonary artery banding led to changes consistent with acute right heart stress or failure with significant increase in heart rate, decreased mean arterial pressure, stroke volume, right ventricular strain, TAPSE, and central mixed venous oxygen saturation. However, significant differences between sepsis and pulmonary artery banding induced hemodynamic changes throughout the experimental period were observed for mean pulmonary artery pressure only (Table 1).

**Table 1:** Variables of systemic hemodynamics, cardiac function and arterial blood gas analysis.

	Baseline		120 min		240 min	
	PAB	sepsis	PAB	sepsis	PAB	sepsis
MAP (±mmHg)	72 (67;80)	70 (61;82)	57 (51;66)	79 (61;92)	34 (30;54)*	54 (44;75)
Heart rate (beats min^-1^)	92 (87;94)	91 (81;102)	114 (107;122)	109* (104;120)	124* (117;140)	115* (108;123)
Cardiac output (l min^-1^)	4.7 (4.2;5.7)	6.4 (5.2;7.2)	4.0 (3.7;4.3)	6.8 (5.9;7.5)^#^	3.3 (2.2;4.5)	5.9 (4.4;7.6)
MPAP (mmHg)	16 (16;18)	17 (14;19)	21 (19;23)	41 (35;44)*^#^	18 (12;21)	42 (29;43)*^#^
Stroke volume (ml)	54 (48;67)	70 (63;77)^#^	33 (31;46)*	61 (45;66)^#^	20 (17;32)*	48 (35;52)*
RV strain (%)	-22 (-20;-31)	-19 (-16;-25)	-14 (-12;-23)	-20 (-18;-23)	-9 (-8;-12)*	-16 (-10;-19)
TAPSE (cm)	1.3 (1.1;1.3)	1.3 (1.1;1.6)	0.9* (0.7;0.9)	1.3^#^ (1.3;1.3)	0.6* (0.5;0.8)	0.7* (0.6;0.9)
SvO_2_ (%)	70 (65;72)	60 (57;63)^#^	51 (41;55)*	58 (40;69)	43 (30;55)*	44 (28;60)
Lactate (mmol l^-1^)	1.1 (1.0;1.1)	1.0 (0.9;1.5)	1.6 (1.5;2.0)	1.6 (1.3;1.8)	1.6 (1.2;2.5)	1.9 (1.7;2.2)*
Hemoglobin (g/dl)	7.9 (7.7;8.0)	7.5 (7.1;7.9)	7.6 (7.2;7.9)	7.4 (6.9;7.7)	7.6 (7.2;7.9)	7.9 (7.2;8.8)

All values: Median (Interquartile Range); PAB: pulmonary artery banding; MAP: mean arterial pressure; MPAP: mean pulmonary artery pressure; RV Strain: right ventricular strain; TAPSE: tricuspid annular plane systolic excursion; SvO_2_: mixed venous oxygen saturation. Generalized linear mixed-effects model with post-hoc comparison to baseline within PAH and sepsis group (*) and pairwise comparison between PAB and sepsis at each time-point (#). Bonferroni correction for multiple testing; p ≤ 0.05.

### Sepsis induces plasma cytokines, markers of endothelial damage and coagulation disorders

**Fig 2:**
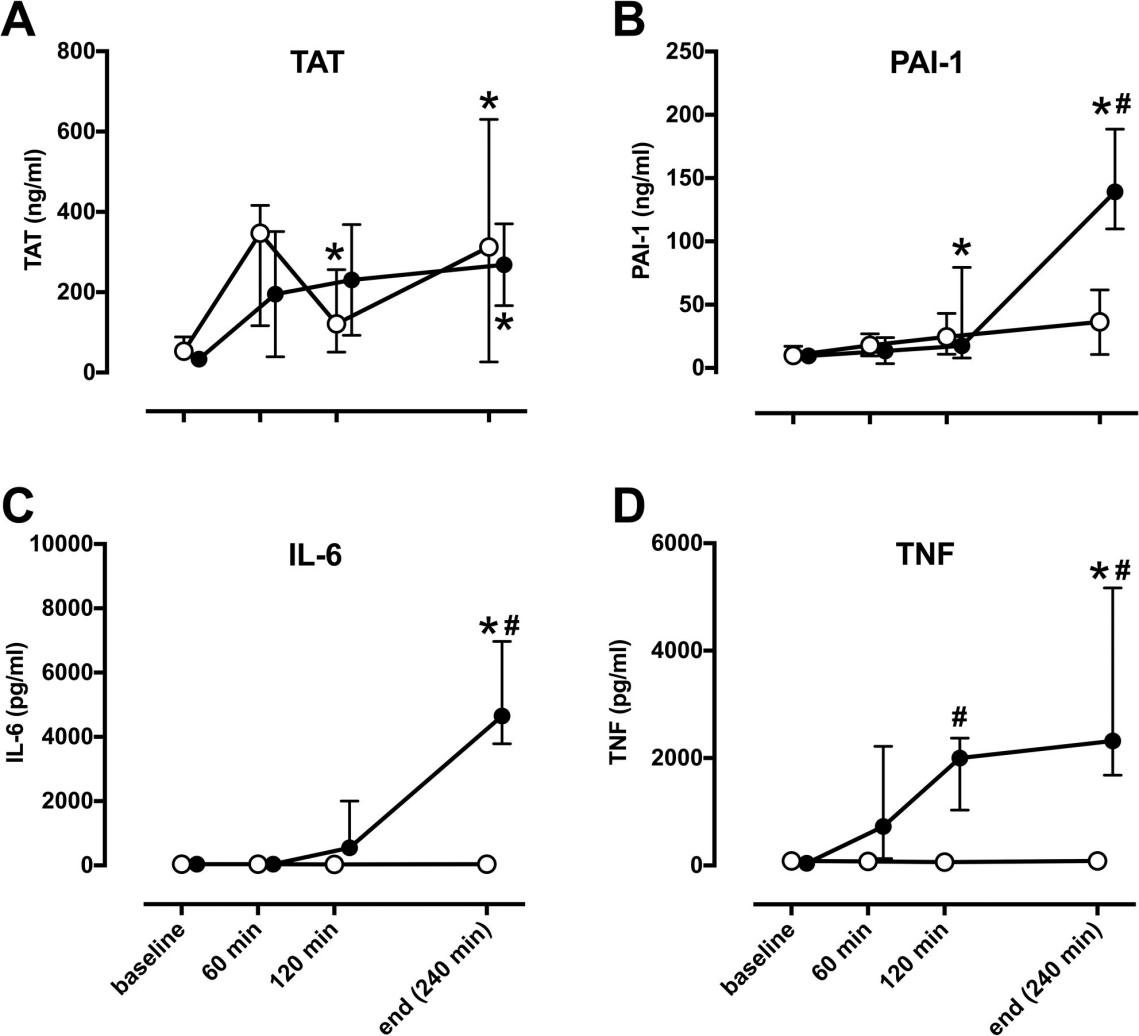
Sepsis but not pulmonary artery banding induces pro-inflammatory changes in plasma. In comparison to baseline, *E*. *coli* induced sepsis (filled circles) significantly increased markers of coagulation TAT (A) and PAI-1 (B) as well as cytokines IL-6 (C) and TNF (D), while pulmonary artery banding (open circles) lead to significant increase of TAT only (*). Significant differences between sepsis and pulmonary artery banding (#) were visible in all markers except for TAT. TAT; thrombin-antithrombin complex, PAI-1; plasminogen activator inhibitor-1, IL-6; interleukin-6, TNF; tumor necrosis factor. All values Median ± Interquartile Range. Generalized linear mixed model with post-hoc comparison to baseline (*) and pairwise comparison between pulmonary artery banding and sepsis at each time-point (#). Bonferroni correction for multiple testing; p< 0.05.

*E*. *coli* infusion led to marked and significant increase of TAT complex and PAI-1 in plasma, while pulmonary artery banding led to significant changes in TAT only (Fig 2). Likewise, cytokines IL-6 and TNF were significantly increased in septic animals only and remained at baseline levels in animals with pulmonary artery banding. The same changes were seen in lung tissue with significant increases in IL-1β, IL-8, TNF and IL-18 and decreases in Cathepsin B and Cathepsin L in septic animals compared to pulmonary artery banding animals (see S1 Fig which illustrates the effect of *E*. *coli* infusion on lung tissue inflammation in comparison to pulmonary artery banding).

### Sepsis induces myocardial cytokine expression

**Fig 3:**
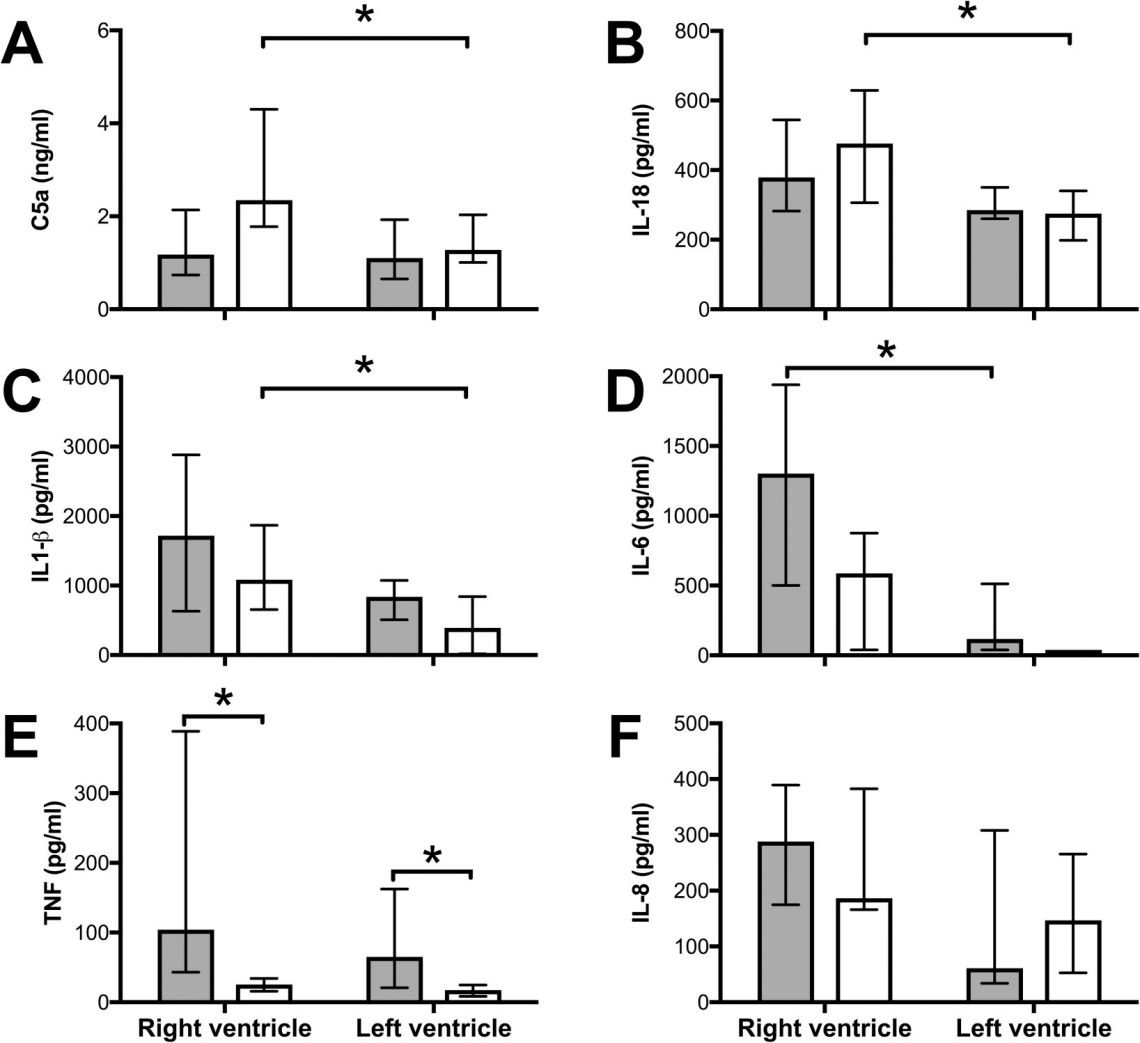
Sepsis and pulmonary artery banding induce inflammation in RV myocardium. Tissue samples from RV and LV at the end of the experiment were analyzed for C5a (A), IL-18 (B), IL-1β (C), TNF (D), IL-6 (E), and IL-8 (F). (A-C) C5a, IL-18 and IL-1β were significantly induced during pulmonary artery banding (open bars) in the RV compared to the LV. (D) IL-6 was significantly increased during sepsis (filled bars) in the RV compared to the LV. (E) TNF was significantly increased in both ventricles during sepsis only and not by pulmonary artery banding, while (F) IL-8 was not significantly different between ventricles nor between treatments. All values Median ± Interquartile Range. Mann-Whitney U test, *; p < 0.05.

Pulmonary artery banding led to significant increase of C5a, IL-18 and IL-1β in RV compared to LV (Fig 3A–3C). Sepsis led to significant increase of IL-6 in RV compared to LV (Fig 3D). TNF was significantly higher in septic compared to pulmonary artery banding animals in both RV and LV (Fig 3E), while no differences were found between septic and pulmonary artery banding animals for the other markers. IL-8 was not significantly changed between treatment groups nor RV and LV (Fig 3F).

**Fig 4:**
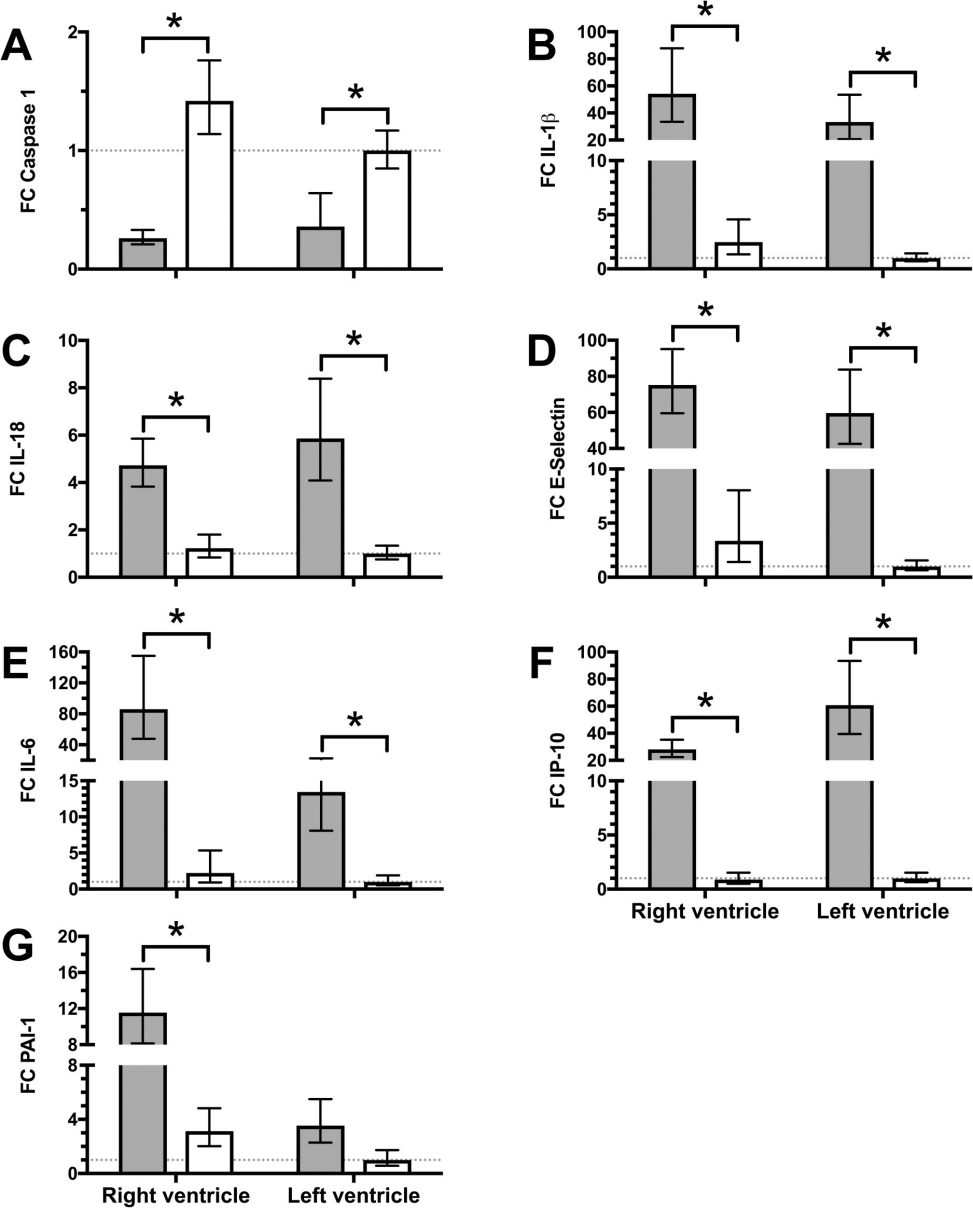
Myocardial RNA-expression differs between sepsis and pulmonary artery banding. Tissue samples from the LV and RV were obtained at the end of the experiment and analyzed for RNA-expression. LV of animals with pulmonary artery banding (open bars) served as control (indicated by dotted line). Sepsis (filled bars) led to a significant decrease of caspase-1 expression (A), while IL-1β increased significantly (B). IL-18, E-Selectin, IL-6, and IP-10 (C-F) increased significantly more during sepsis compared to pulmonary occlusion in both RV and LV. PAI-1 (G) did only increase significantly in RV during sepsis compared to pulmonary occlusion. No statistically differences were found between ventricles during sepsis or pulmonary occlusion. FC; Fold Change, all data presented as Mean ± 95% confidence interval, 1-way ANOVA with post-hoc all pairwise comparison and Bonferroni correction, *; p < 0.05.

RNA expression in myocardium showed significant differences between septic and pulmonary artery banding animals in the RV for all seven markers investigated (Fig 4); in septic animals, downregulation of Caspase 1 was accompanied by upregulation of IL-1β, IL-18, E-selectin, IL-6, IP-10 and PAI-1. Likewise, expression of pro-inflammatory RNA in the LV was significantly increased in septic animals compared to pulmonary artery banding animals in all investigated markers except for PAI-1.

### Cathepsin L plays a protective role in heart function

**Fig 5:**
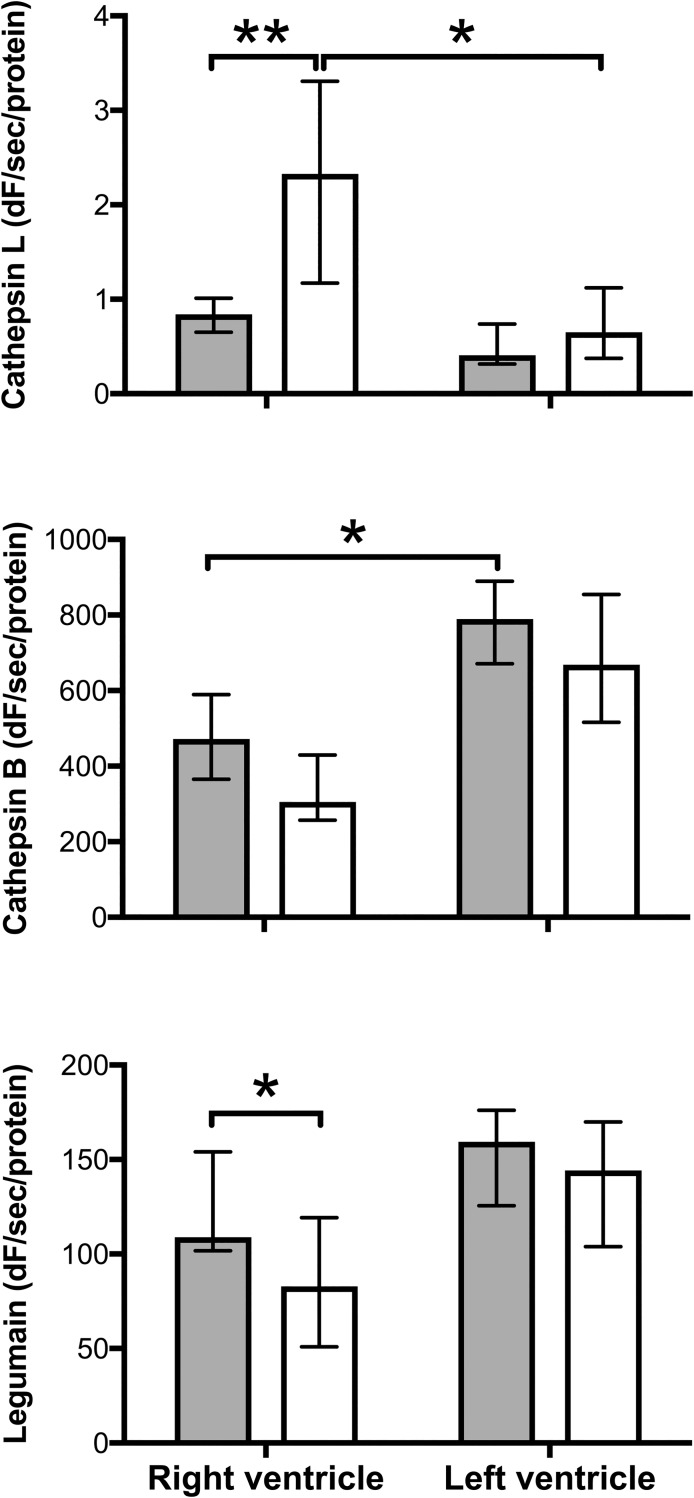
Pulmonary artery banding causes increased activity of cathepsin L in RV myocardium which is prevented by sepsis. Tissue samples from LV and RV were homogenized and enzyme activities of cathepsin B, L and legumain were measured with fluorescent peptide substrates. Solid bars are from septic animals and open bars from pigs with pulmonary artery banding. Cathepsin L activity (A) was significantly increased by pulmonary artery banding but not by sepsis. Cathepsin B activities were generally slightly lower in RV (B) and pulmonary artery banding caused reduced legumain activity in RV when compared to sepsis (C). All values Median ± Interquartile Range. Mann-Whitney U test, *; p < 0.05.

Analyses of the lysosomal cysteine protease family are found in Fig 5.

## Discussion

In this porcine study, *E*. *coli*-induced sepsis caused significant increases in pro-inflammatory cytokines in both RV and LV myocardium compared to a control group with pulmonary artery banding. The stress on RV and the clinical presentation were comparable in both groups.

The challenge of a failing RV in the course of sepsis has been recognized for a long time [24][25] and is regarded a common event during septic shock [20][7]. In hyperdynamic circulatory shock, RV myocardial dysfunction is seen in sepsis, but not in trauma [13], and may represent disease severity and hence worse clinical outcome [11]. In a former study, we used the same 10 animals with *E*.*coli* induced sepsis and showed significantly impaired cardiac function evaluated by echocardiography [17]. In the present study, pulmonary artery banding induced isolated RV impairment. It caused similar substantial reductions in cardiac output, stroke volume and central venous oxygenation as did sepsis. Cardiac function was decreased to at least comparable or even greater extent in pulmonary artery banding, compared to sepsis animals. However, in contrast to sepsis, pulmonary banding did not lead to systemic inflammation. Furthermore, we observed increased pro-inflammatory markers in lung parenchyma of septic animals, but not in animals undergoing pulmonary artery banding. Only septic animals had a significant rise in mean pulmonary artery pressure, whereas it was normal in pigs with pulmonary artery banding. The mechanism for PAH in porcine sepsis can to a large extent be explained by large amounts of macrophages in the porcine lung [16]. In the septic animals, right ventricular pressure was a backward effect of a global contraction along the whole pulmonary artery tree. Animals with the very proximal pulmonary artery banding developed similar backward-caused right ventricular pressure, but the pressure in the pulmonary artery distal to the banding was, as expected, much lower.

The mechanisms leading to RV dysfunction in sepsis are poorly understood [26]. In septic patients, endothelial dysfunction measured by plasma endothelin-1 has been shown to be associated with RV dysfunction [27] and E-Selectin with hypotension [28]. Heart specimens are hardly ever examined. Thus, animal models have been used to elucidate the pathophysiology of myocardial dysfunction in sepsis. In pigs, myocardial depression in sepsis has been attributed to alterations in calcium handling in combination with decrease in mitochondrial respiration [29]. The cause for the myocardial dysregulation is still unclear, but inflammatory signaling through ICAM-1 [30], TNF and IL-1β [31] has been shown to decrease cardiomyocyte function in rodents [9]. Here, in a porcine model we show that, indeed, protein levels of TNF, and IL-6 as well as mRNA levels of IL-1β, IL-18, IL-6, IP-10 increased in septic animals only and are thus not due to PAH and consecutive RV impairment alone. Endothelial activation markers PAI-1 and E-Selectin mRNA levels increased in septic myocardium only, indicating endothelial dysfunction and active leucocyte recruitment in sepsis but not during the comparable mechanical stress of pulmonary artery banding. PAI-1 has recently been linked to acute innate immune response in sepsis [32], and might be a predictor of mortality in sepsis [33]. Myocardial E-Selectin increase corresponds to findings of E-Selectin as a specific marker for sepsis-induced hypotension in patients [28]. Interestingly, Caspase 1 mRNA levels decreased in septic myocardium. This phenomenon was first described in blood cells from septic patients, resulting in defective IL-1β production [34]. Defective IL-1β production might explain the relatively low increase in protein IL-1β in comparison to IL-1β mRNA in myocardium of septic animals observed in our study.

In humans, it is speculated that RV failure in sepsis is due to the rise in pulmonary artery pressure and an increased cardiac demand. If this was the only cause, one would expect to see the same and even aggravated changes in RV failure due to exclusive RV afterload increase *e*.*g*. in pulmonary embolism, arrhythmia, or pulmonary artery banding.

In the present study, pulmonary artery banding led to higher C5a, IL-18 and IL-1β protein levels in right compared to left ventricle. C5a is a potent anaphylatoxin involved in a myriad of pro-inflammatory processes. It is formed during the cleavage of C5 in the complement system cascade and is involved in many cardiac disease states [35]. In heart failure, the complement system has been shown to get activated and then returns to normal upon therapy with *e*.*g*. left ventricular assist device [36] or cardiac resynchronization therapy [37]. This indicates that the C5a activation seen in the present study is indeed indicative of acute right heart failure. IL-1β and IL-18 both belong to the IL-1 superfamily and recent evidence shows that IL-1β activates IL-18 in the course of myocardial ischemia/reperfusion injury and acute heart failure [38]. Both exert negative inotropic effects independently of each other [39]. Taken together, the activation of the innate immune system in the present study demonstrates that acute -mechanically increased RV pressure leads to a different pattern of immune response in the right ventricle, compared to sepsis-induced PAH. Future studies should evaluate if generation of NO through the iNOS system or reactive oxygen species generation in general is affected in the same manner.

The lysosomal cysteine protease family comprises 11 cathepsins and the asparaginyl endopeptidase legumain [40][41]. Although primarily localized to lysosomes and involved in intracellular protein turnover, these enzymes are also secreted and have extracellular regulatory functions *e*.*g*. in controlling extracellular matrix and growth factors. In the present study, the ubiquitously expressed endopeptidase cathepsin L in pigs with pulmonary artery banding was significantly higher in the RV compared to LV. These findings support the hypothesis that cathepsin L plays a protective role in heart function. Overexpression of human cathepsin L in murine hearts has been shown to improve cardiac function and to inhibit hypertrophy, inflammation and fibrosis [42]. Knock-out mice deficient in cathepsin L have shown increased mortality in the course of myocardial infarction [43].

In comparison, cathepsin B is thought to have an overall negative effect, demonstrated in a study where inhibition of cathepsin B reduced the activation of caspase-11 and inflammasome [44]. Legumain is a cysteine protease related to caspases and important in the activation and processing of cathepsins [45].

In the present study, only cathepsin L levels were significantly different between the two groups. These findings may imply that in the course of sepsis the protective induction of cathepsin L is ablated, while as in our study there is no similar effect on the negative form cathepsin B or the regulator legumain. Cathepsins have not yet been widely investigated in sepsis and their effects are therefore not known. However, our results tempt the speculation that observed effects on extracellular matrix, growth factor and cytokines could be attributed to differential cathepsin activity.

## Conclusions

We demonstrate in this porcine study that mechanically-increased right ventricular pressure leads to a different myocardial inflammation compared to a sepsis-induced PAH. The prominent PAH seen in porcine sepsis should therefore not exclude porcine models in sepsis research. Evaluation of targeted anti-inflammatory therapies with the aim to reduce inflammation in the heart and thus prevent cardiac failure in the course of sepsis could therefore be evaluated in porcine models.

## Supporting information

S1 Table Detailed information about pigs (Sus scrofa) used in accordance with ARRIVE guidelines. (DOCX)

S1 Fig The effect of E. coli infusion on lung tissue inflammation in comparison to pulmonary artery banding. (TIFF)

S1 Dataset Underlying data for all tables and figures. (XLSX)
