# Supplementary material for: Sepsis causes right ventricular myocardial inflammation independent of pulmonary hypertension in a porcine sepsis model
Source: PLoS One. 2019 Jun 27;14(6):e0218624. doi: 10.1371/journal.pone.0218624 (PMC6597071; doi:10.1371/journal.pone.0218624)
Supplement: S1 Table — (DOCX) [file pone.0218624.s001.docx]

**Supplementary Digital Content 1**

## Supplemental table 1: detailed information about pigs (*Sus scrofa*) used in accordance with ARRIVE guidelines [1]

| **Item** | **Explanation** |
| --- | --- |
| Species and strain | *Sus scrofa*, NOROC |
| Weight | 52 ± 3 kg |
| Age | 5 to 6 month old |
| Sex | 4 males : 12 females |
| Origin | Open-air farm (Fredrikstad, Norway) |
| Health status | No signs of infections (no diarrhoea, no infected scratching marks) |
| Housing | Pig boxes (2.4 sq.m) with raised floors in a 40 sq.m room |
| Bedding | Aspen bedding (B&K Universal Ltd, Hull, UK) |
| Housing atmosphere | Tightly regulated room temperature (20**°**C) and humidity (55.7%), as well as 12 hours light/dark cycles. |
| Feeding | Unlimited access to commercial pig feed and tap water |
| Acclimatization period | One day and night |
| Transport to OR | Sedated by intramuscular injection of ketamine (1500 mg), azaperone (160 mg), and atropine (1 mg) in the cage. An intravenous catheter was established in an ear vein and sedation deepened using pentobarbital (0.5 – 1 mg/kg). |
| OR atmosphere | Sterile and stable environment with regulated temperature (22°C) and humidity (50-60 %). |
| Temperature | Controlled with heating blankets to 38°C arterial temperature. |
| Anaesthesia | Balanced anaesthesia with Isoflurane 0.6-1.5% end tidal concentration and morphine 0.1-0.3 mg kg^-1^ h^-1^ infusion. Anaesthesia depth was regularly evaluated by no reaction to sharp hoof and nose pinching. |
| Respiration | Standard respirator (Leon, Heinen Lowenstein, Bad Ems, Germany). Fixed tidal volume (18 ml/kg), positive end-expiratory pressure (5 cm H_2_0) FiO_2_ (0.45), resulting in mean airway pressure of 10 ± 0.5 cm H_2_O. |
| Hemodynamic monitoring | Central venous access via the external jugular vein and placement of pulmonary artery catheter (Edwards Lifesciences, Irvine, CA). Artery catheters (8 Fr introducers) placed in both carotid arteries for artery pressure measurement, blood sampling and insertion of micromanometers (Millar Instruments, Houston TX). |
| Euthanasia | Bolus of Pentobarbital (300 mg), Morphine (10 mg) and Potassium chloride (50 mmol) |

OR: operation room, iv: intravenous

**References**

1. Kilkenny C, Browne WJ, Cuthill IC, Emerson M, Altman DG (2010) Improving bioscience research reporting: the ARRIVE guidelines for reporting animal research. PLoS Biol. 8:e1000412 doi:10.1371/journal.pbio.1000412
