# Supplementary figures and images for: Sepsis causes right ventricular myocardial inflammation independent of pulmonary hypertension in a porcine sepsis model
Source: PLoS One. 2019 Jun 27;14(6):e0218624. doi: 10.1371/journal.pone.0218624 (PMC6597071; doi:10.1371/journal.pone.0218624)

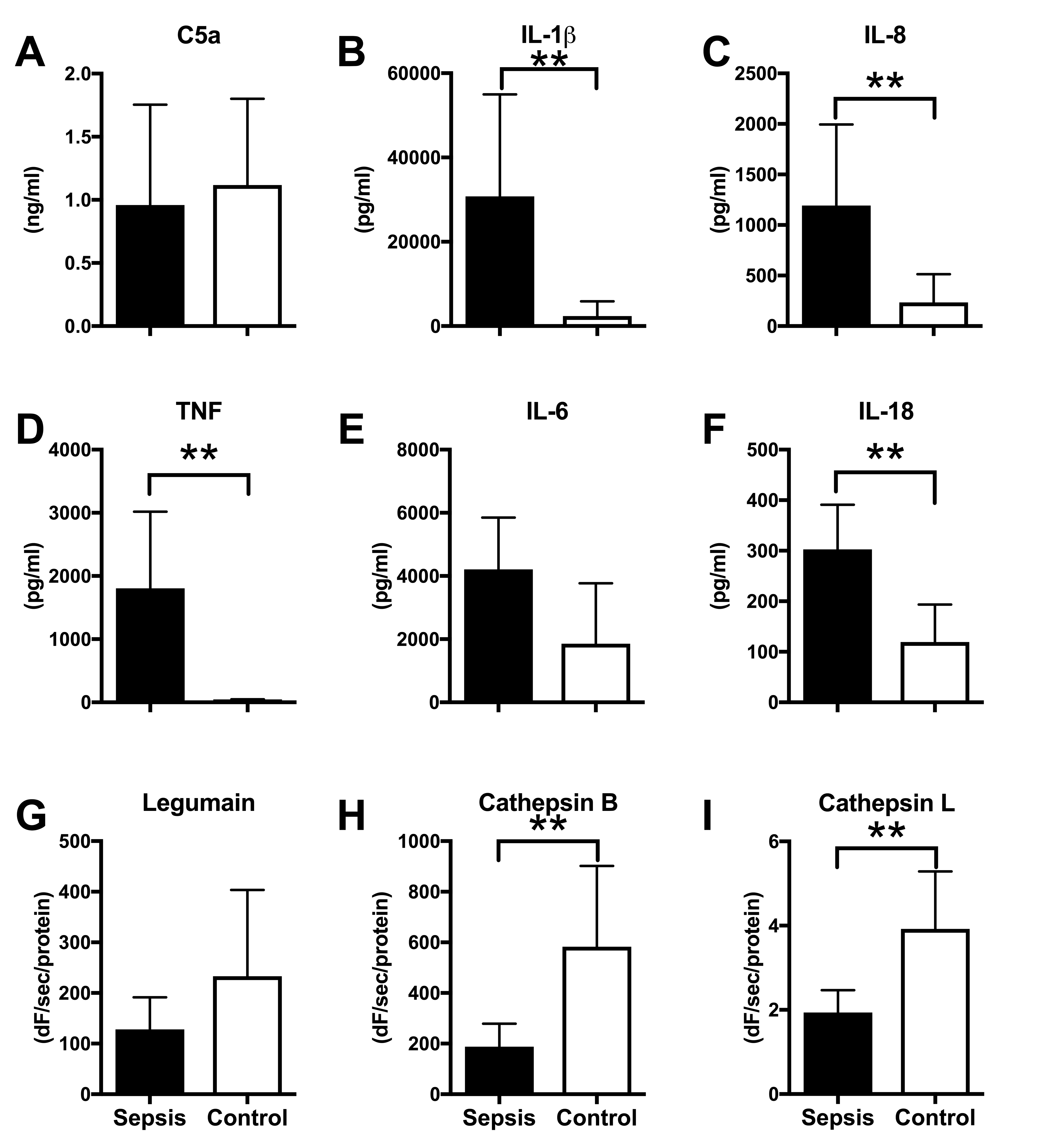

Supplement: S1 Fig — (TIFF) [file pone.0218624.s002.tiff]
